# Assessing the Primary Data Hosted by the Spanish Node of the Global Biodiversity Information Facility (GBIF)

**DOI:** 10.1371/journal.pone.0055144

**Published:** 2013-01-25

**Authors:** Javier Otegui, Arturo H. Ariño, María A. Encinas, Francisco Pando

**Affiliations:** 1 Department of Zoology and Ecology, University of Navarra, Pamplona, Navarra, Spain; 2 Unidad de Coordinación de la Infraestructura Mundial de Información en Biodiversidad (GBIF) en España, Real Jardín Botánico, Madrid, Spain; CSIR-Institute of Microbial Technology, India

## Abstract

In order to effectively understand and cope with the current ‘biodiversity crisis’, having large-enough sets of qualified data is necessary. Information facilitators such as the Global Biodiversity Information Facility (GBIF) are ensuring increasing availability of primary biodiversity records by linking data collections spread over several institutions that have agreed to publish their data in a common access schema. We have assessed the primary records that one such publisher, the Spanish node of GBIF (GBIF.ES), hosts on behalf of a number of institutions, considered to be a highly representative sample of the total mass of available data for a country in order to know the quantity and quality of the information made available. Our results may provide an indication of the overall fitness-for-use in these data. We have found a number of patterns in the availability and accrual of data that seem to arise naturally from the digitization processes. Knowing these patterns and features may help deciding when and how these data can be used. Broadly, the error level seems low. The available data may be of capital importance for the development of biodiversity research, both locally and globally. However, wide swaths of records lack data elements such as georeferencing or taxonomical levels. Although the remaining information is ample and fit for many uses, improving the completeness of the records would likely increase the usability span for these data.

## Introduction

### The Biodiversity Crisis

There is a general agreement on the hypothesis that biodiversity is in crisis [1]–[5]. Continuous losses in ecological quality of different areas of the globe suggest that we could be in the midst of the sixth major extinction event in the history of Earth [6]. There is also a wide consensus in that a large enough volume of quality data is mandatory in order to effectively undertake studies aimed to solve these global issues [7]–[12]. These ‘global studies’ require a change in both the scope and the way of getting data [13], as well as the development of proper methodologies to transform volumes of data into actual knowledge, one of the main challenges in biology [14].

Much ecological research is based on Primary Biodiversity Data (PBD) [15], also called Primary Biodiversity Records (PBR) when forming part of a database. A PBD is a piece of information detailing an occurrence: the sighting or sampling of an individual belonging to a species on a specific moment and place [16], [17]. In other words, a PBD describes (in its most basic form) what has been observed or collected, and where and when it was. Additional data may enhance this basic triplet: the more access we have to biodiversity primary information, the better conclusions we can get from those works in terms of reliability (see for example [18] or [19]).

In local-scoped studies, a researcher may afford to obtain direct samples for data, either in the field or through museums and herbaria. However, in global-scoped studies, this becomes overwhelming [20]. Thus, data facilitators become extremely useful for collecting data for global studies [21]. A data facilitator is an initiative (institution, database, or project) that links different data sources in a common frame, in order to allow easy access to the whole data set through a single gateway.

### The Global Biodiversity Information Facility

The Global Biodiversity Information Facility (GBIF, http://www.gbif.org/) is now the largest initiative of this kind [22], [23]. GBIF was proposed by the Organization for Economic Co-operation and Development (OECD) ‘Mega Science Forum Working Group’, and was formally established by Governments in 2001 with the aim of “making the world’s primary data on biodiversity freely and universally available via the Internet” [24]. Through a global network of 57 countries and 47 organizations, GBIF “promotes and facilitates the mobilization, access, discovery and use of information about the occurrence of organisms over time and across the planet”. Technically, the facility works, among other things, as a biodiversity information aggregator and, at the time of writing, it enables access to more than 317 million primary biodiversity records made available by 342 institutions, hereinafter data publishers, from a common data portal (http://data.gbif.org/). GBIF is headquartered at Copenhagen, Denmark, and has a decentralized structure with national and regional nodes [25].

Data publishers are at the core of GBIF. They are, among others, research centers, universities or biodiversity information networks who keep a collection of data and make them publicly available [26]. Each publisher shares one or more data sets, also called data resources, containing individual records – the actual PBDs – and metadata such as e.g. ownership, intellectual property rights, information from a sampling campaign or data concerning a particular taxonomic group. PBDs are shared using a normalized data standard (Darwin Core, DwC) to which fields in the source database are mapped [27]. Metadata about the resources are published through the GBIF data portal by using one of several resource sharing mechanisms, e.g. through harvesting by the DiGIR, or TAPIR communication protocols or direct publishing through the Integrated Portal Toolkit (IPT) [28] into the GBIF Metadata Catalogue (http://http://metadata.gbif.org/catalogue/). It is important to keep in mind that data publishers are the ultimate owners of their data and their rights, and any concern on data quality is therefore responsibility of the publisher of those records [29], GBIF acting as an indexing and discovery mechanism [30].

### GBIF.ES, the Spanish Node of GBIF

Spain, being one of the founding members of GBIF, has now signed the new Memorandum of Understanding (accessible at http://www.gbif.org/orc/?doc_id=2955) which gives continuity to the GBIF network in Spain indefinitely. Back in 2002, the Science and Technology Ministry commissioned the Spanish National Research Council (CSIC) to create and maintain, with the support of the Royal Botanical Garden and the Natural Sciences National Museum, a Coordination Unit that would manage GBIF’s activities in Spain, the Spanish National Node of GBIF [31].

GBIF.ES works as a network of Spanish biodiversity information institutions, in the role of a proxy through which Spanish research centers can make their biodiversity data publicly available online. Currently, this comprises 62 institutions and projects, 58 of which make use of the GBIF-Spain Hosting Service. It is the data hosted there that we are analyzing in this paper. In order to enhance and improve Spanish data-based research, GBIF.ES also has its own data portal (http://www.gbif.es/datos/) allowing direct querying of the resources it hosts and indexes, including images of “taxonomic grade” as defined by Ariño and Galicia [32]. In addition, GBIF.ES also hosts a mirror of the main data portal.

### Aims

In order to explore to what extent could the Spanish node of GBIF help improving global and regional biodiversity research, we decided to assess the GBIF.ES-hosted Primary Biodiversity Records in search for patterns, strengths, and weaknesses in the biodiversity knowledge it may contain. Of particular interest to us was assessing whether these patterns may impact the fitness-for-use [33] of the primary data published by GBIF.ES.

A second objective was to build on and update previous reports where partial aspects of GBIF.ES contents were tackled, using smaller or earlier subsets of data or other metadata of interest, such as collections data [34], [35].

## Materials and Methods

Through an agreement with the GBIF Secretariat, we were able to directly mine the full GBIF database in its June 2011 state, instead of using the data portal which is impractical for this particular task. Nevertheless, the records we accessed are identical to those accessible through the data portal.

GBIF indexes are organized in a large MySQL database containing several dozen tables. Central to the database is the table of occurrences, containing all PBR contributed by publishers. We set up a MySQL database and queried the GBIF index by using SQL statements, extracting all data related to GBIF.ES as data publisher.

From all the available field sets that are included in the occurrences table, we focused on those, which are most centrally related to PBD: geospatial (explicit location and coordinates), temporal (year, month, and date of occurrence), and taxonomic information, as well as some additional metadata (data describing the records, such as data type or data resource to which the record belongs) from the publisher and the resources.

We further reorganized and queried the selected subset with scripts written for MySQL and FoxPro and data models in Access. Summary data were generally processed in Excel. Our strategy was to aggregate, organize, compile, and represent the information in such ways so as to observe departures from the isotropic pattern that a random distribution of data would yield, following the general approximations proposed elsewhere [34]–[39]. This also allowed us to detect errors in data, often as outliers from the main data body.

Higher-level taxonomic information for each record may be provided from the original dataset or automatically assigned by GBIF when records are indexed. When interpreting taxa, GBIF routines attempt to check each record against a taxonomical backbone largely constructed atop Catalogue of Life [40]. However, when publishers supply a taxon string that cannot be interpreted against the backbone (for example, spelling variations or different taxonomies) a new string is added to the backbone. We constructed our taxonomic tree for the dataset by first retrieving the taxon concept treatment of each record from the taxonomical backbone when available. Next, null (unavailable) concepts were resolved whenever possible by checking the lowest available taxon name against the unified taxonomic tree in Catalogue of Life (CoL). If that failed, higher levels were attempted by searching in the constituent databases in CoL and other available databases such as Species 2000 (Index Fungorum (http://www.indexfungorum.org/names/names.asp), WoRMS (http://www.marinespecies.org/), AlgaeBase (http://www.algaebase.org/), Fauna Ibérica (http://www.fauna-iberica.mncn.csic.es/) or ITIS (http://www.itis.gov), among others. A manual review of records resistant to the above treatments was made to locate misspellings or taxon level misplacements, and these were corrected and the records re-checked as above. The remaining taxon strings not fit after all the above procedures were manually searched in specialized literature if they corresponded to more than 0.02% of the entire dataset. When the available taxonomy in the literature did not match the CoL taxonomy, an attempt was made to reasonably place the taxon within the CoL tree. If all failed, the corresponding taxon levels were left blank.

## Results

### Metadata

The July, 2011 GBIF.ES-hosted databases contained 5,166,998 primary biodiversity records in 139 data resources belonging to 59 institutions (see table 1). As of December, 2011 GBIF.ES hosted 152 resources and 5,209,796 records. Four other Spanish publishers further contributed 16 resources and 724,092 records. The largest data resource – the ‘Anthos’ Plant Information System – contributed 1,109,506 records.

**Table 1:** Volume and completion of the different aspects of primary biodiversity information accessible through GBIF.ES.

Feature	Value (%)
data resources	139
records	5,166,998
records with coordinates	3,769,746 (72.96)
records with country	5,053,060 (97.79)
records with year	3,130,867 (60.59)
records with date	2,278,819 (44.1)
records with kingdom	4,793,406 (92.77)
records with taxonomy	4,462,555 (86.37)
records with data type	5,166,998 (100)

**Figure 1:**
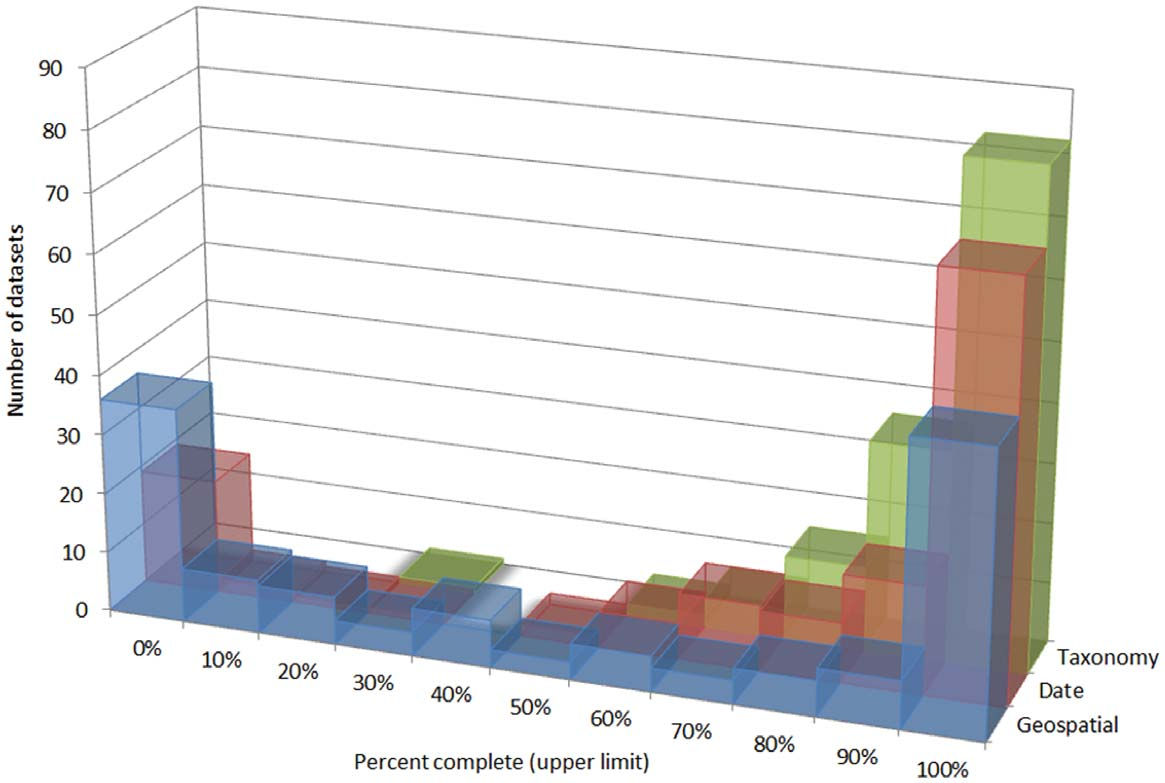
Summarized completeness of the different data resources linked through GBIF.ES. Bars represent the number of resources publishing a certain percent of their data records complete (i.e., containing information in the relevant fields) for three aspects of primary biodiversity data.

The degree to which records contain complete PBD-related information varied (figure 1). One third of data resources lacked entirely coordinates while another third had all records complete. Complete date references (year+month+day) were fully recorded in 40% of data sources, but there were about 20 resources which did not report fully-qualified dates. However, locality and year were routinely present. On the other hand, most records had their country, year, and kingdom fields filled (table 1).

**Figure 2:**
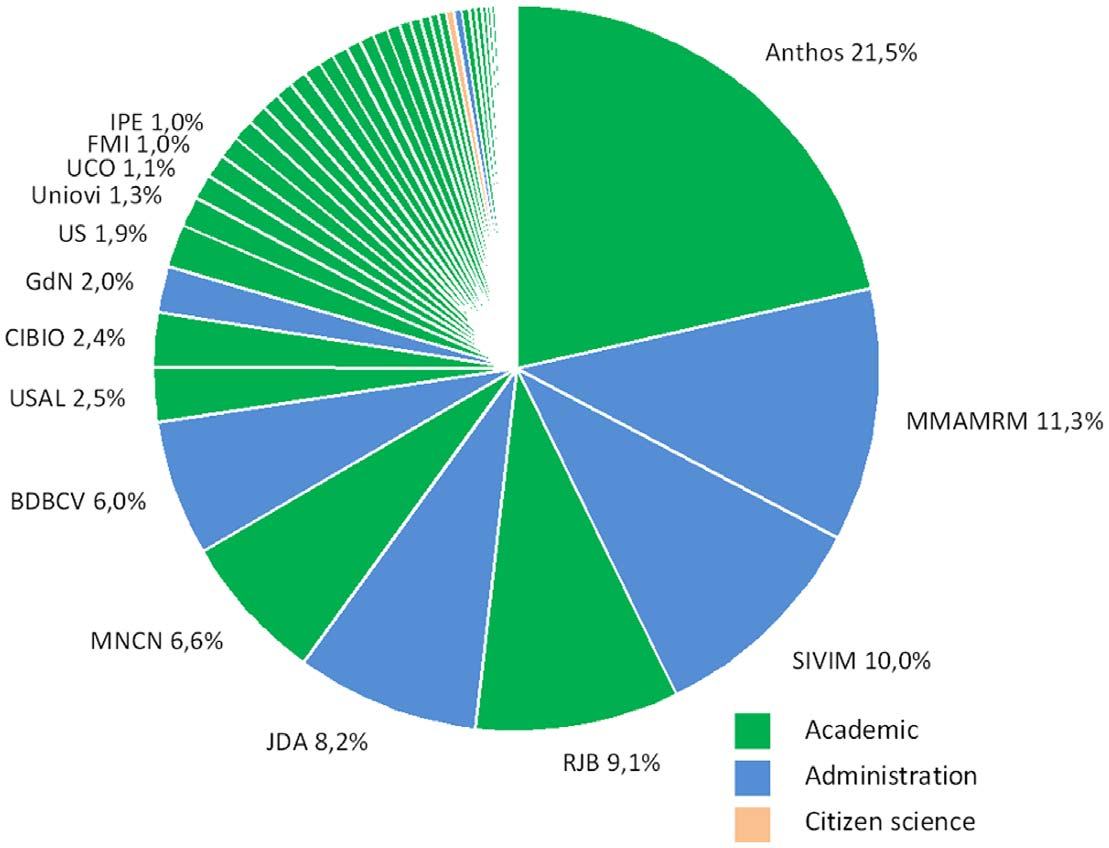
Relative volume of records published by each institution through GBIF.ES. (An institution may be publishing more than one resource). Color indicates the type of institution.

Fourteen resources (10%) accounted for 75% of the records (figure 2): Anthos-Sistema de Información de las plantas de España (Fundación Biodiversidad, Real Jardín Botánico-CSIC); SIVIM-Sistema de Información de la vegetación Ibérica y Macaronésica; Cartografía de vegetación a escala de detalle 1∶10.000 de la masa forestal de Andalucía; Inventario Nacional de Biodiversidad 2007: Aves (Ministerio de Medio Ambiente, y Medio Rural y Marino. Dirección General de Medio Natural y Política Forestal); Vascular Plant Herbarium (MA, Real Jardín Botánico, Madrid); Banco de Datos de la Biodiversidad de la Comunitat Valenciana; MNCN-ICTIO (Museo Nacional de Ciencias Naturales, Madrid); Herbario SALA (Universidad de Salamanca); Catálogo Florístico Histórico de Navarra (Gobierno de Navarra); Inventario Nacional de Biodiversidad 2007: Mamíferos (Ministerio de Medio Ambiente, y Medio Rural y Marino. Dirección General de Medio Natural y Política Forestal); CEUA, Instituto de Investigación CIBIO (Universidad de Alicante); Herbario SEV (Universidad de Sevilla); MA-Fungi (Real Jardín Botánico, Madrid); and Flora Mycologica Iberica.

**Figure 3:**
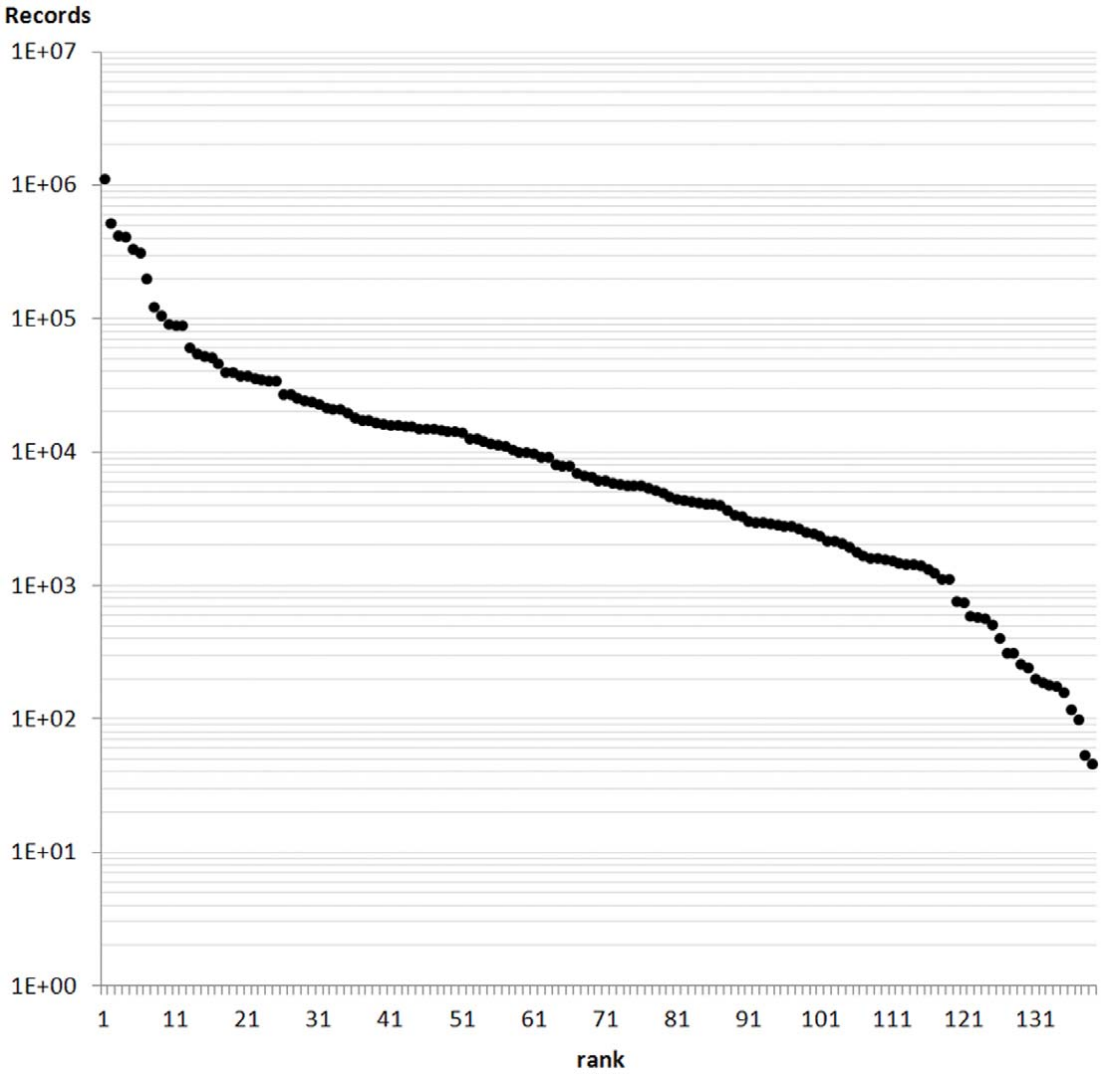
Whittaker plot of the number of records (log scale) against their order by size. The flat distribution has a low dominance but no canonical abundance distribution could be fitted.

The ranked log of data records in resources showed a relatively flat Whittaker plot (figure 3), suggesting low dominance (Simpson’s D = 0.08; Shannon’s H’ = 3.29). None of the four classical distribution models (geometric, logarithmic, log-normal, or McArthur’s broken stick) could be successfully fitted to the data.

**Figure 4:**
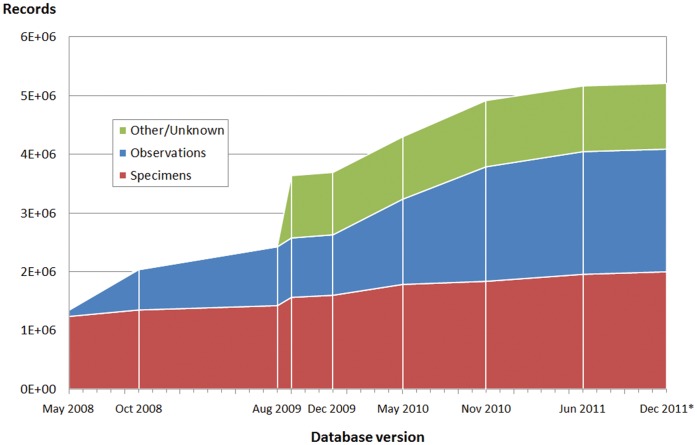
Cumulative volume of records in successive versions of the index by data type. In most cases, data are incorporated in discrete chunks, consistent with the acquisition of new resources rather than updates in existing ones.

Specimen-based data represented an estimated 40% of the records by the end of 2011, while observations accounted for about 38%. Information on the basis of record was lacking for most of the remaining 22%, except for about 0.2% of living specimens (tissues and germplasm). However, the observation-based data had been steadily increasing over successive versions of the database, while specimen-based data had grown comparatively less (figure 4). A few data publishers had incorporated vast amounts of data which had not been declared as belonging to one or the other category, although it was assumed that most of these new datasets were recording observations or compiling observations from other sources. Therefore, the proportion of observational data in the publisher could potentially have grown from less than 8% in 2008 to more than 60% in 2011.

### Geospatial Information

**Figure 5:**
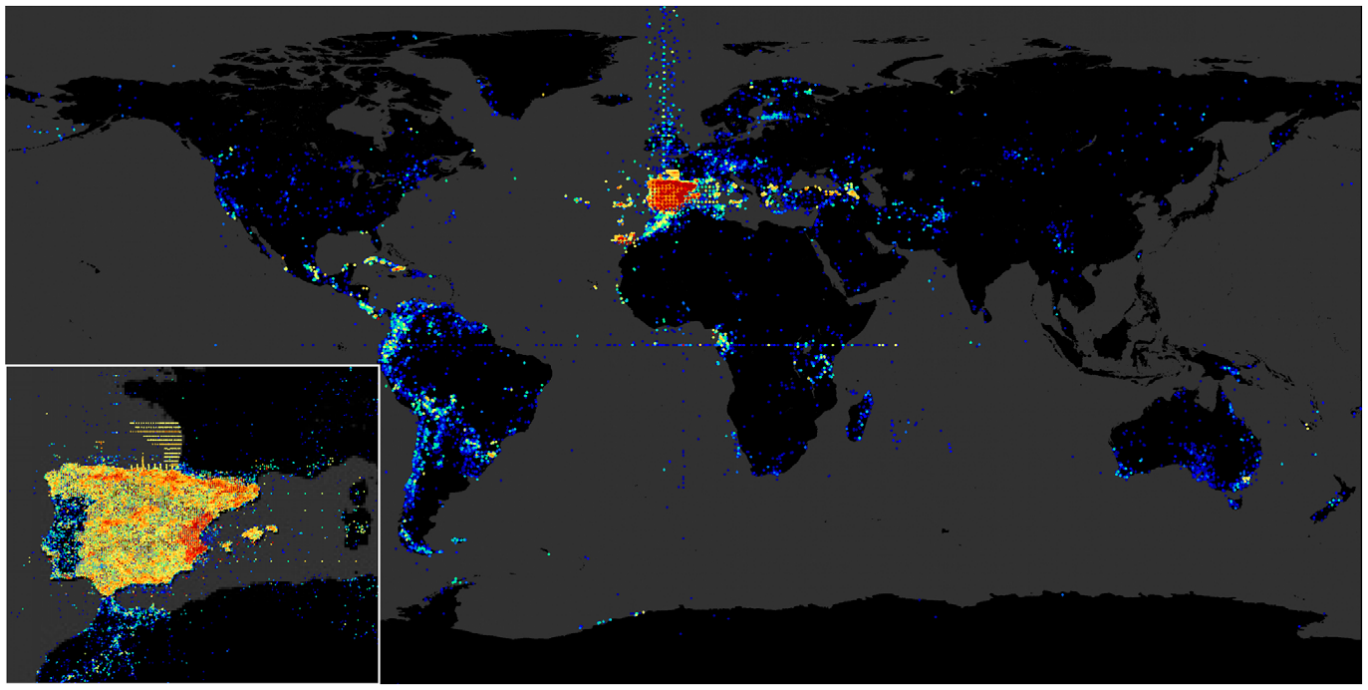
Visualization of the geospatial information offered through GBIF.ES. Each point is a unique latitude/longitude pair. Color represents the binary log of record density for that point from blue (minimal) to red (maximal).

**Figure 6:**
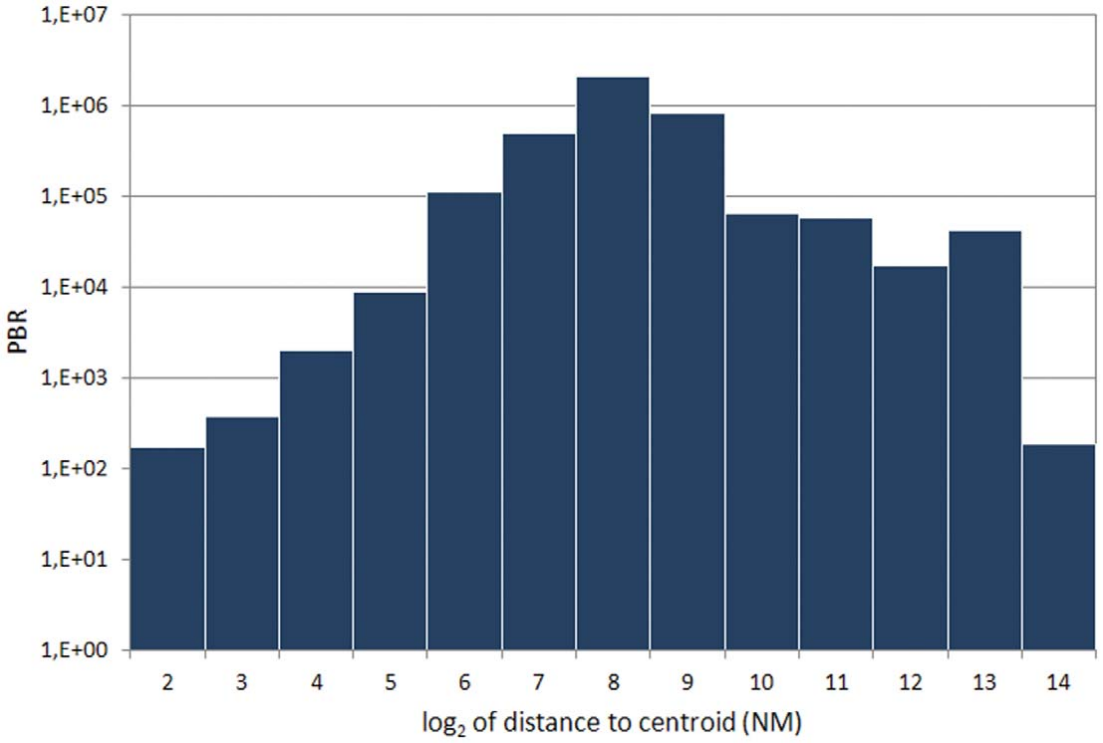
PBD volume according to the log of the distance to the centroid of all data. The units are Nautical Miles (NM) and the centroid is located near Toledo, Spain. The bimodal histogram has two modes at approximately 460 km (approximate radius for the Iberian Peninsula) and 7400 km (distance range to America).

Most records published through GBIF.ES were recorded in the Iberian Peninsula and Macaronesia, with 87.9% of the records declaring ‘ES’ (Spain) as country of collection. Records providing coordinates represented 73% (3.77 million, table 1) of the datasets, and are projected in figure 5. There was also a significant number of records coming from Central and South America, Europe and the Mediterranean, Western Sahara, and Equatorial Guinea. The centroid for records declaring coordinates was at N 39.392, W 3.584, not surprisingly fairly close (80 km) to the Iberia Peninsula geographical centroid. The distribution of records according to the distance to this centroid was clearly bimodal on a log-log scale (figure 6), with most records located within peninsular Spain and a second block between 5000 and 8000 NM from the centroid.

Some patterns visible in the map were identified as artifacts arising from georeferencing mistakes, such as the latitudinal lines of data leaving the Iberian Peninsula along meridians, or imprecise georeferenciation, such as an equidistant net of high-density points patterned along UTM zones or the coarse sexagesimal grid, or erroneous nullification of coordinate components, such as lines along the equator or (less conspicuously) along the prime meridian. Other striking patterns were the high data densities within the boundaries of the Valencia region (East coast) and along the Pyrenean range, and a series of latitudinal lines across the Bay of Biscay; however, we related these patterns to actual availability of data from specific data publishers (see Discussion).

### Temporal Information

**Figure 7:**
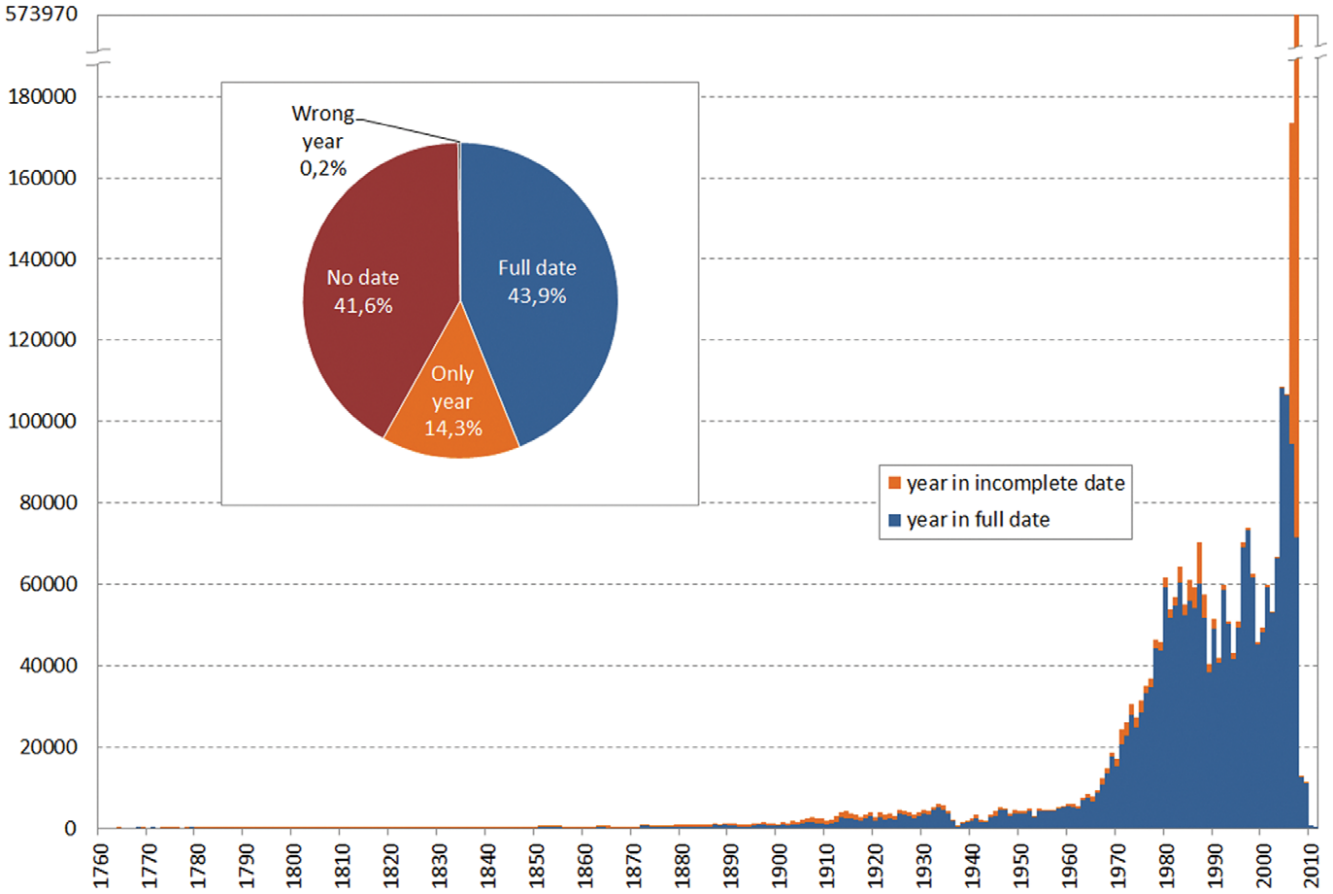
Number of records collected/oserved each year as declared in the record. The year information was obtained from completed date fields (blue) or from the year field in incomplete dates (orange). Inset: Distribution of records according to whether they had complete or incomplete dates. There is a large number of records that report year zero (i.e., missing).

The records published through GBIF ranged three centuries, from roughly 1750 to the current year, but only 44% were attributed to a specific date, with a further 14% being given just year of collection (figure 7, inset). Although a small number of dated records claimed a future collection date, or were dated many centuries back, these were apparently dating or digitizing errors. Most records declared collection dates from 1965 onwards (figure 7), with 2007 being the single year claiming nearly 11% of all dated records. However, most records in the latter year had incomplete dates (only year information): 78.8% of all incomplete records were declared as collected on 2006 and 2007, and belonged to one single institution (Ministry of Environment, Rural and Marine Affairs) sharing two main resources (National Inventory of Biodiversity: Birds and Mammals). The number of records declined very sharply afterwards, and even though the latest database examined in full was compiled in June 2011, the number of records collected from 2008 onwards was very small as compared with the preceding decades.

**Figure 8:**
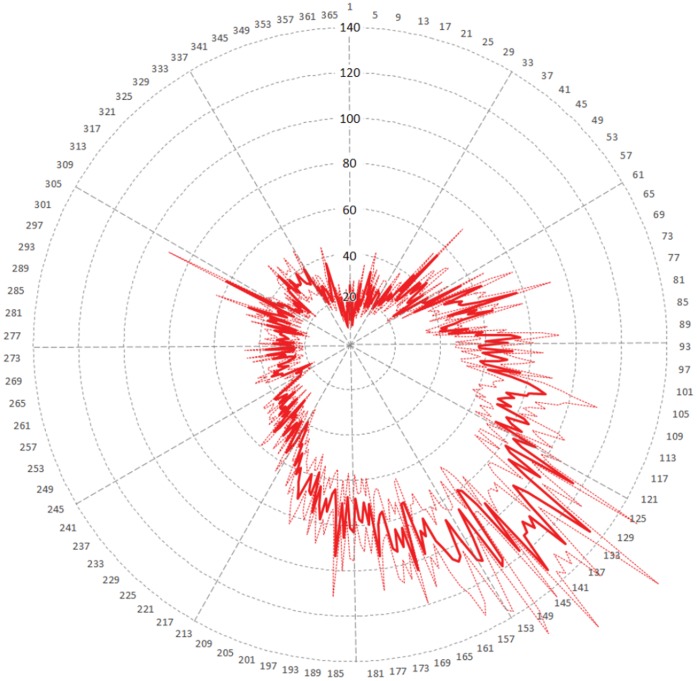
Average distribution of records within years. Year rank is from 1750 to 2011. Solid line represents average number of records and dotted lines represent standard error of the mean. Radii mark the first day of each month. Day of year is numbered 1–366; non-leap years lack day 60. There is a high record bias towards spring/summer.

Examination of the seasonal pattern revealed that datasets published through GBIF.ES contained records collected mostly in late spring and early summer (figure 8). This very strong pattern was spread across many datasets and was consistent year through year, as revealed by the relatively low standard error of the means. Secondary, local maxima appeared in late fall and belonged to a number of fungal collections. Minima were located around the turn of the year, in winter.

**Figure 9:**
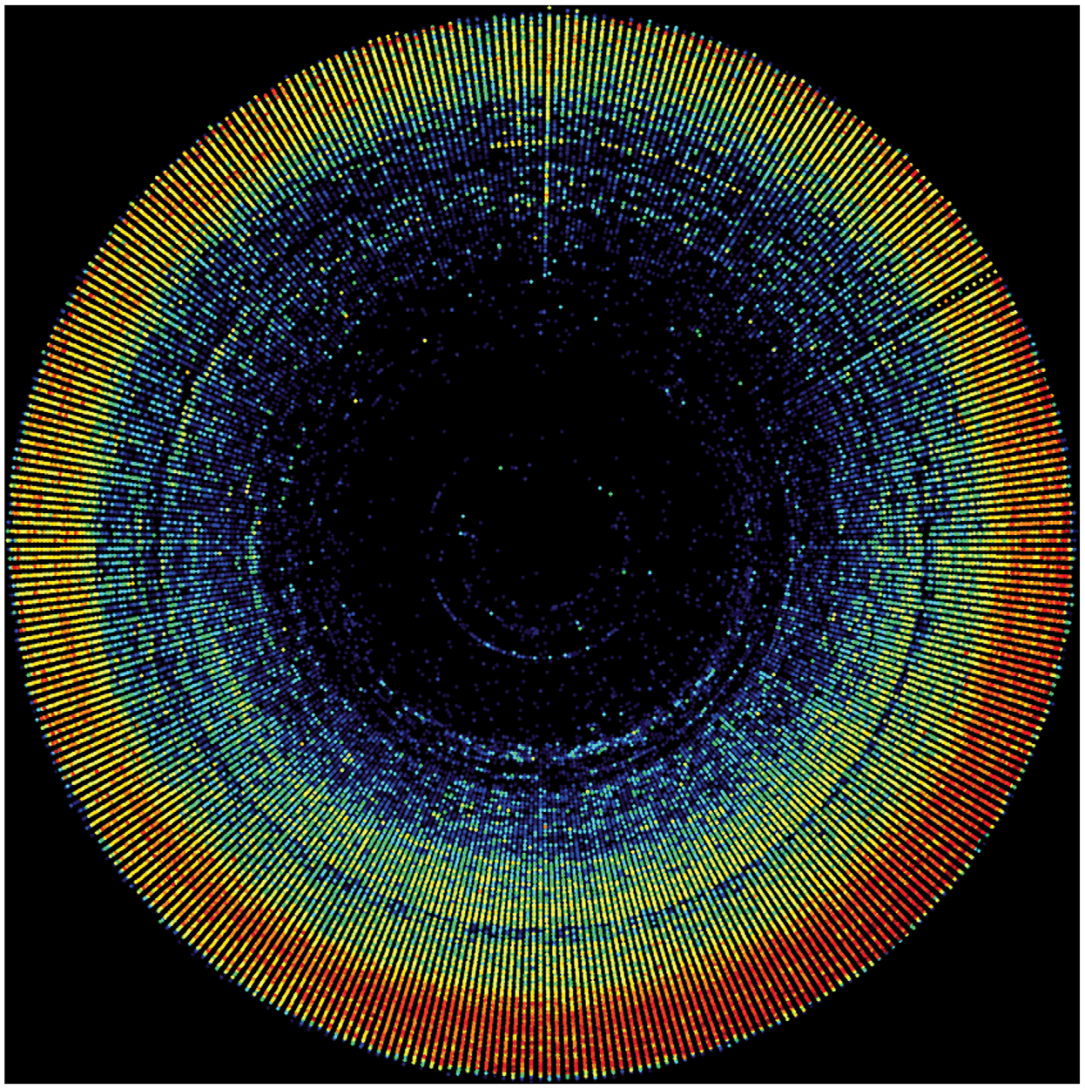
Chronhorogram of the records shared through GBIF.ES. Each point is a day of the year in polar coordinates where the radius is the year and the angle the day of year. Center (origin) is 1750 and January 1st is top. Color represents the record density for that day (point) on a log(2) cold-hot scale. The seasonal summer patterns are observable, as well as non-periodic phenomena such as th low-density ring representing little data during the Spanish Civil War period. The vertical spoke corresponds to dates given as January 1st for want of a true date within the year instead of blank day/month.

The chronological and seasonal evolution of the data gathering can be displayed in a chronhorogram [34], [37] (figure 9). Some obvious features here are the exponential growth in data availability towards recent years, as well as the consistency of the summer pattern across years.

### Taxonomic Information

**Figure 10:**
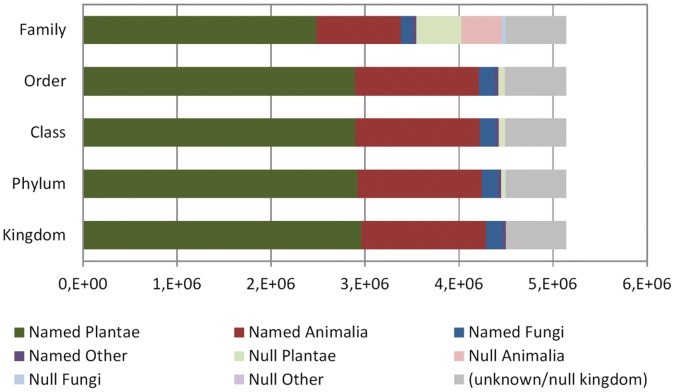
Number of records in each kingdom having taxonomical information declared by the publisher. Lighter shades indicate records for which such information was missing at each taxon level. Gray: Records that had not been assigned a kingdom.

Publishers may or may not have declared taxon ranks above the mandatory species name. Approximately 7.2% of the publishers did not declare what kingdom each of their records belonged to. About 86% of all records had information in other high taxon levels (order, class, phylum/division), but family data was missing from nearly one third of records (table 1). For those records with known, declared kingdom, small differences were detected among kingdoms as regards to missing phylum and class data (animals were 100% complete while plants were 98% complete). Order completion was very similar (98% and 99%), but family data completeness varied: plants’ families were more often recorded (84%) than animal families (68%) of fungi families (73%). Chromista, Protozoa and Bacteria had nearly all their upper taxonomic levels complete (figure 10).

**Figure 11:**
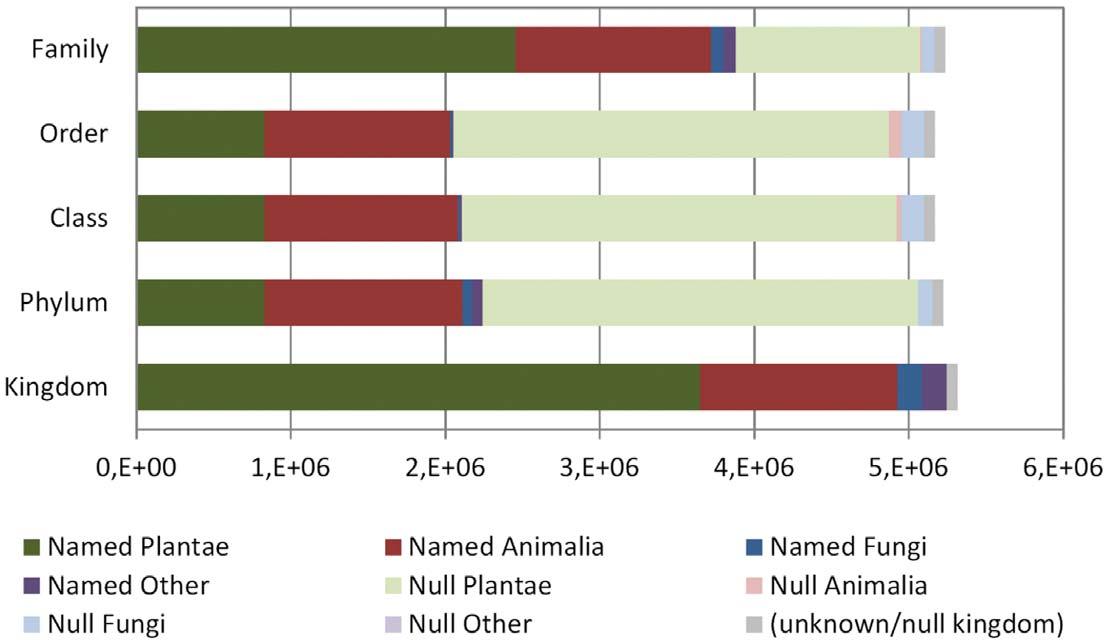
Number of records in each kingdom having taxonomical concept information supplied by GBIF taxonomical backbone. Taxonomic placement is given according to the interpretation of the taxon at the lower levels (genus, species epithet, and infraspecific ranks). Lighter shades indicate records for which such information was missing at each taxon level. Gray: Records that had not been assigned a kingdom. Kingdom was assigned according to the Species-2000 based backbone but many records lacked intermediate levels for the Plants and Fungi kingdoms.

Taxonomic placement is provided by GBIF for the records it serves, based on an automated taxonomical backbone using CoL taxonomy. This attribution, executed at indexing time, was apparently able to locate most of the missing kingdoms, and after this treatment 97.6% of records were served with a kingdom concept even though the publisher might not have done it. Animals were also attributed successfully to other high-level taxon concepts, as only about 2% of the records in the kingdom were left without phylum, 6% were missing class, and less than 1% was not assigned to a family. However, plant and fungal records fared poorly in higher taxonomic levels. Only 23% of plant records were located in the backbone above the order level even though two-thirds (67.3%) had a family name assigned by the attribution procedure. Fungal had even worse attributions, with about one half being attributed family but only 7% order or class (figure 11).

In order to understand the taxonomical structure of the data, a new set of attributions was attempted by validating records against the CoL database and other databases (see Methods), followed by a series of manual parsing and checks against taxonomical literature to correct misspelling, wrong taxon attributions, etc., based in provided families and taxon names for species. Approximately 287,000 distinct taxon strings existed in the dataset, which were reduced to 182,000 strings belonging to 3838 families. These families were manually attributed to higher taxonomical levels to remove the nulls in the taxon fields and therefore examine the taxonomical distribution of the dataset.

**Figure 12:**
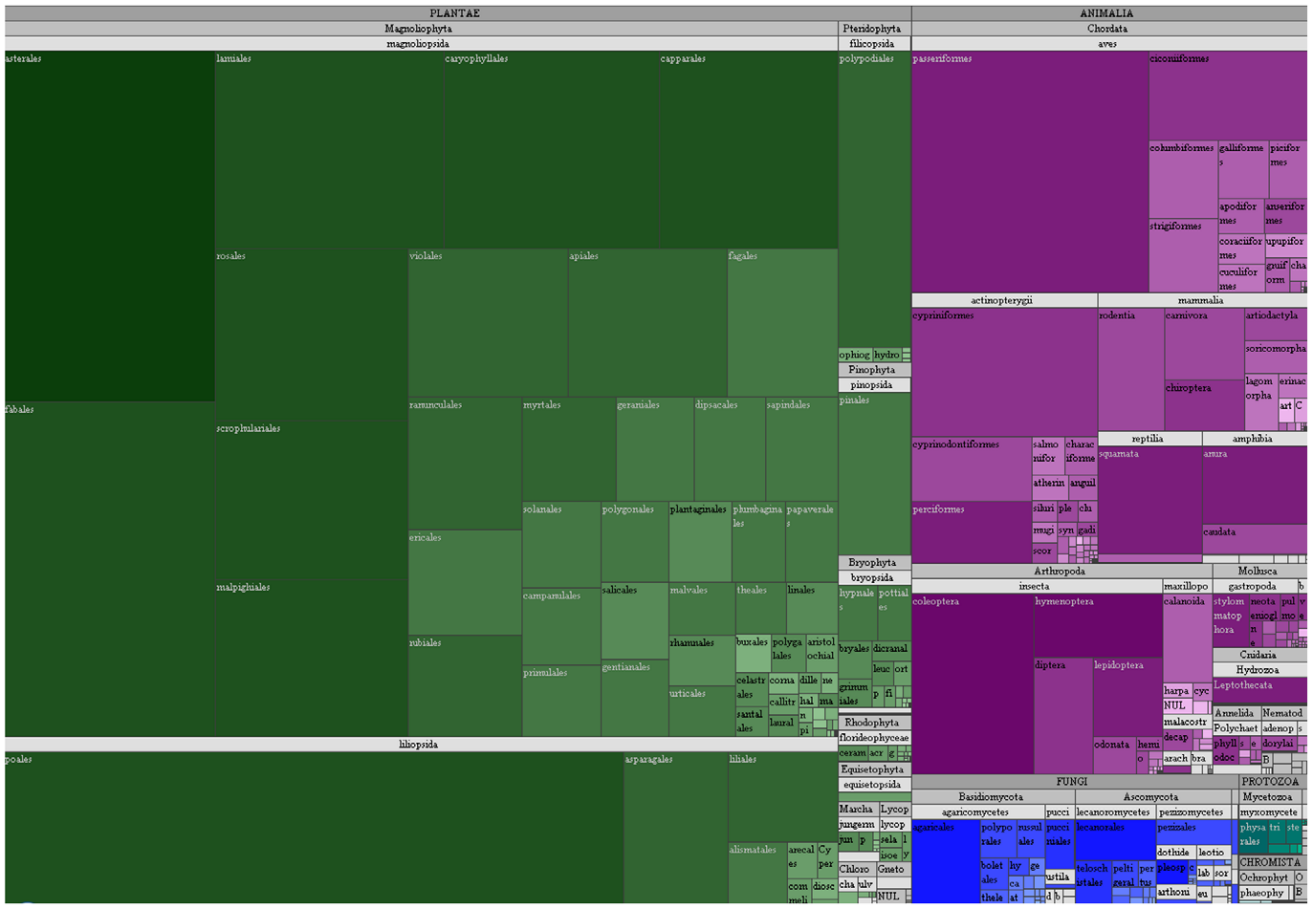
Nested surface map (tree map) of the taxonomy of GBIF.ES. Each cell belongs to a taxonomic order and is nested into a class cell; this is nested into a phylum/division cell, which is nested into a kingdom cell. The area of the cell is proportional to the number of records. Color hue represents kingdom. Color shade is proportional to the binary log of the number of different taxon strings (i.e., combinations of taxon level-specific names from kingdom to species) supplied by the providers for each order. (Darker is more).

Most records published through the GBIF.ES Hosting Service corresponded to species of plants (69.6%). About one quarter (26.0%) were animals, and 3.6% fungi. Other kingdoms accounted for the remaining 0.8%. Among plants, Poales, Asterales, and Fabales were the best represented orders, while passerine birds were the most recorded (figure 12). The distribution treemap shows a database dominated by Angiosperms and Vertebrates; together they represented almost 83% of all records. Among fungi, both Basidiomycota and Ascomycota were very similarly represented.

The number of records seemed to be approximately proportional to the number of different taxon strings used by the publishers to refer them. The color density of the treemap is proportional to the log of the number of different strings identifying the taxon, where the string was constructed from the kingdom, phylum/division, class, order, family, and species name in each record. Asterales was a particularly rich plant order as compared to others, while the coleopteran and hymenopteran insects and perciform fish were among the richer animal orders.

## Discussion

### Metadata

As of June 2011, GBIF.ES was the tenth largest data publisher in the entire GBIF Network, and indeed the largest data publisher among the Spanish and Portuguese participants, representing almost 1.8% of the total volume of GBIF records. Its largest data set – the ‘Anthos’ Plant Information System – was the thirty fifth largest data resource among 12,712 data resources. By January 2012, large data networks, mainly bird observations, had also been indexed and GBIF.ES became the 14th publisher, accounting for 1.6% of the total volume. Among the data-publishing GBIF nodes, GBIF.ES is currently eight in terms of volume.

Many basic PBD-related fields such as country, year or kingdom are largely complete. However, finer details such as geographical coordinates and especially fully-qualified dates are often missing. 20 collections show no record with completely qualified dates while the records of 43 resources lack coordinates. Such data-deficient resources might be hard to improve, at least harder than those partially complete, since they could represent sampling campaigns with no associated georeferencing, or perhaps second-hand data.

Although such “incomplete” records may be fit for certain purposes [33], such uses are also limited. For instance, time-related information that is not dependent on the period of the year (i.e., inter-annual distribution changes) may not need exact dates, and are therefore fit for that particular use while they may be unfit to observe, for example, changes in migration start patterns. Time-dependent problems may be inherent to the digitization process, that is exact dates not recorded, or be introduced because of interpretation, parsing, or date treatment problems at compile time [41].

The change in the type of shared records (figure 4) may be related to the historical availability of data (the “low hanging fruit” effect [42]). Specimen data curated in botanical gardens and zoological museums were incorporated first, as these were often already in digital form as a part of the normal data keeping mechanisms. As GBIF and its National Nodes became aware of the data richness observational resources could offer, the focus moved into that area. Thus, observational data came in later, but because records of this kind are easier to produce, they have been growing at a much faster rate. There are resources that do not specify what type of records they are sharing, or that incorporate newer types (for instance, sound recordings or live material), or, very often, that hold several types of records. In their report on 2009 data, Encinas and Pando [35] found a mere 17% of observation-reporting sources, but these already accounted for 64% of records. The current 2011 data reduce the percent of observational data to slightly over 50%, but this results from a more detailed analysis where non-observational, non-specimen based data have been separated. Purely specimen-based data have increased remarkably less than other types of data since 2009, suggesting the effective incorporation of the “ready” collections data. But there has been a steady increase nonetheless, also suggesting that the existence of a “long tail of data” [43] is being exploited or slowly discovered and incorporated [44].

**Figure 13:**
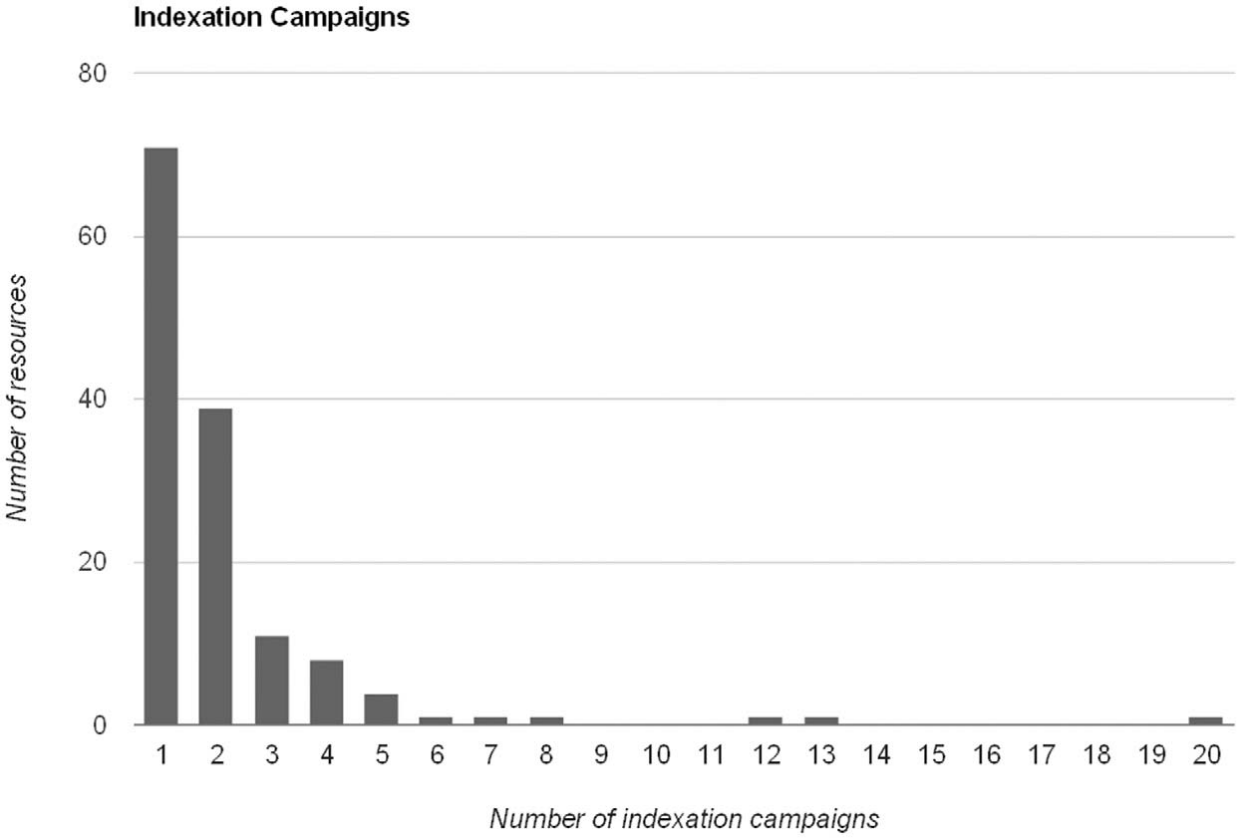
Number of times a data resource has added new records to its body (indexing campaigns). Bars show the amount of data resources that have added records X times, where X is the value of the abscissa. Most of the data resources add data once (at the moment of creation), and in most cases, once more. It is unusual for a resource to add records more than 4 times.

Most observed changes are related to new resources as opposed to updated data. Very few resources updated their record numbers once created (figure 13), and even less added records more than 4 times from their incorporation. Most increases in the volume of records shared by the publisher were due to new resources. Encinas and Pando [35] proposed that the rate of accrual was highly dependent on the taxonomical type of the data in the resources being incorporated: botanical data were largely published first at a steady pace and started to increase the rate from 2007 onwards, while zoological data started later and are yet to increase rate. The overall rate of increase shows steep changes where entire new resources are incorporated [35].

In a heterogeneous set of data collating many different sources, an obvious question is whether observed trends spread across components or result from a particularly dominating component showing the trend and imposing it over the entire ensemble. GBIF.ES has a relatively low dominance among data sources. The largest resource accounts for less than one-quarter of the total records published (figure 2), and therefore general trends observed are likely representative of GBIF.ES.

### Geospatial Information

The bias in geospatial information is primarily linked to the national nature of the publisher. Most records come from Spain and Portugal, with a second block of data collected in Spanish-speaking countries elsewhere around the world. This is not surprising when the Spanish Scientific Expeditions of the 18th and 19th centuries to the Americas are taken into account. However, most records were also collected in recent times (see Temporal patterns), thus indicating that the bimodal structure of the dataset as regards to geolocation may be actually related to current trends, such as scientific international collaborations among researchers from Spanish-speaking countries.

Examination of the geometrical patterns in the data as plotted on the map (figure 5) readily yielded at least seven remarkably “strange” patterns: regular latitude increments, low precision in coordinates and inverse longitude, bias due to system conversion, latitude and/or longitude equal to zero, regular geometric pattern, political boundaries and uniform coordinate shifts. The last two patterns are not geometric and may go less noticed, but can be noted as well. All of them could be isolated and tracked to very specific causes, and while some revealed digitization issues, others had valid, underlying causes.

1. Homogeneous latitude increments

Lines of terrestrial data unrealistically extending along meridians northward from mainland Spain apparently changed in homogeneous increments the latitude data. While we cannot ensure what could be the cause here, one common problem when preparing blocks of data in a spreadsheet, for example Microsoft’s Excel, is that certain copy/paste procedures may be used by mistake that increase numerical in a field values as they are copied to new lines (e.g., dragging a numerical cell while holding down another key).

1. Low precision in coordinates and inverse longitude

The uniform, high-density matrix of evenly-spaced dots reveals that coordinates were recorded or published at low precision. One possibility is storing the coordinates without any decimals, or rounded up to the nearest degree which would render a pattern centered in the degrees. A striking feature in the pattern is that for certain providers, the matrix continues east to a maximum longitude that matches the maximum western longitude. One common problem here would be that the western longitudes have not been negated as required in the decimal degree format, therefore becoming positive (east) longitudes when they should be negative (west).

1. System conversion bias

The sloping lines going approximately north-south with an angle in the middle of Spain can be tracked down to the commonly used military grid (UTM-based) coordinate system in Spain for presence data, where presences are recorded as just the UTM square cell (at the chosen resolution degree) where the observation was made. Converting UTM references to lat/long coordinates may entail deciding which coordinate best represents the UTM cell, often without provision for the necessary definition of coordinate uncertainty [45], [46]. As UTM zones slant at the contact point, the resulting pattern is a set of tilted grids.

1. Latitude and/or longitude equal to 0

This is a classic effect of an interoperability issue: for a particular database, latitude 0 could mean “no latitude given”, but when merged with other data sources, 0 is not the same as null. The result shows in the map as a very visible line at zero latitude, and a fainter one at zero longitude, both suggesting that there are many records that use 0 instead of leaving the latitude field empty. In a coordinate, zero is a valid datum and should not therefore be used in lieu of null.

1. Regular geometric pattern

The last striking, geometrical pattern is a series of latitudinal lines in the Bay of Biscay extending from the French shoreline westwards. These were tracked down to a single resource (a plankton survey), and are no artifacts but simply the parallel transects followed by the research ship.

1. Political boundaries

Data density increases sharply along the borders of the administrative unit of the Valencian Community (east of Spain). This is typical of a local resource (often an administration) gathers lots of records from a given area having political boundaries, obscuring the real data density unless some type of sampling effort can be factored in. Such phenomena severely hamper the usability of presence data for ecological studies without extensive rationalization when more than one region is analyzed at once.

1. Uniform coordinate shifts

The last pattern shows a set of points directly west of the southeast tip of the Iberian Peninsula. Closer examination reveals that they belong to terrestrial data suppliers, and that the clusters of the data points resemble the number and shapes of the Canary Islands. In fact, these are indeed data that if edited for latitude minus ten degrees, would fall directly on such islands. Therefore, it is very likely that they represent a georeferencing or coordinate transcription mistake.

### Temporal Information

**Figure 14:**
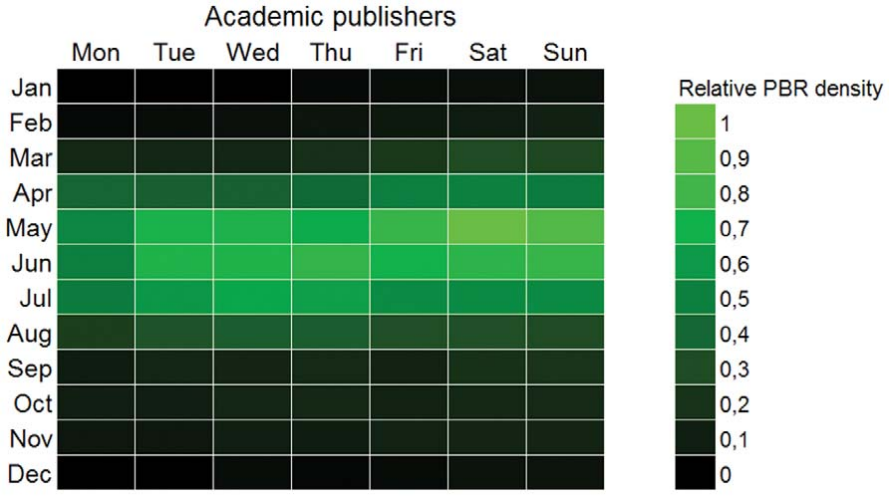
Hebdoplot of academic publishers. Record density map for days of week and month for records published by academic institutions. Color scale is proportional to the binary log of records. Brighter color is more records. The pattern for these data, often collected by scientists, academicians or amateurs, is denser in and around the weekends and very seasonal for late spring-early summer.

**Figure 15:**
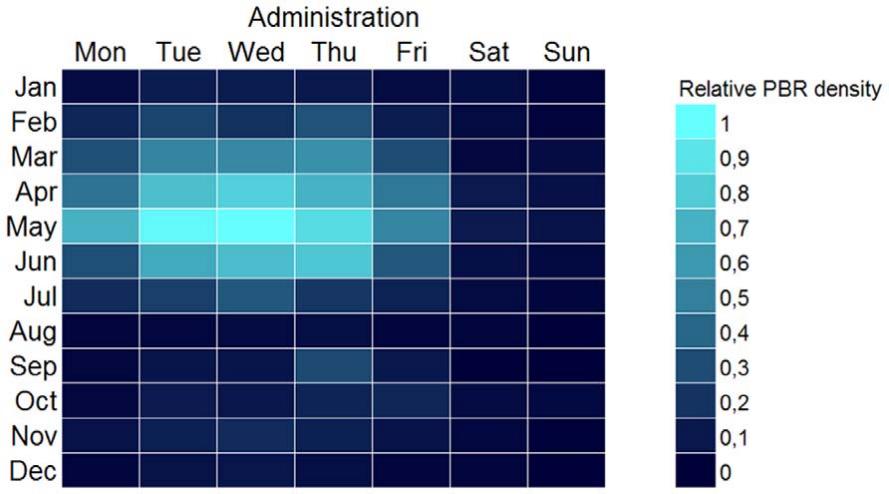
Hebdoplot of administration publishers. Record density map for days of week and month for records published by government agencies and other administration. Color scale is proportional to the binary log of records. Brighter color is more records. The pattern is denser in weekdays, away from weekends. The seasonal pattern maximizes in May and drops to near zero in around the summer vacation month (generally August in Spain).

The seasonal pattern revealed by the chronhorogram [33], [37] could either be a natural pattern associated to the growth season, or a sampling bias: researchers may tend to prefer sampling in summer rather than in winter. Anecdotal evidence may support the second hypothesis. By reorganizing all the data in a weekly calendar form, one may try to find a pattern appearing along an unnatural but human cycle (week). Such pattern would be associated to human activity but not to natural phenomena. Should this pattern appear, its mere existence would cast doubt on supposedly natural patterns also subject to human activity such as seasonal activity. If data from all providers are summarized together, there is no weekly pattern. However, when separating academy institutions from administration bodies as suppliers of data, a distinct pattern emerged: many data records gathered by academic institutions or individuals working in academia were collected during weekends (figure 14), while administration- and government-dependent institutions concentrated in work days (figure 15). Saturdays in May appeared to be the favorite sampling/observation period in the academic or scientific realm. We may conclude that sampling bias exists, and that is therefore a strong possibility to help explaining seasonal patterns. Other anecdotal evidence gleaned from the cronhorograms are the data gap during the Spanish Civil War (1936–1939) when most science halted, or the set of imprecise PBR that were recorded as January 1st for any particular year for which the exact sampling date was unknown, showing as a vertical line of dots. Weekly patterns appear as spirals.

In addition, the common mistake of using “0″ (zero) as equivalent to absence of value in a field also applies to the ‘year’ field, which should be null when unknown. We found more records with actual zero than records with any value from 1990 to 2005 in their ‘year’ field, as it happened with the coordinates of some records. But now, it is easier to detect this as an issue, since it is extremely unlikely for an occurrence to have been taken at year 0.

### Taxonomic Information

The treemap in figure 12 describes the actual distribution of records in the taxonomical tree and the variety of taxon strings, but it is not an accurate representation of the taxonomy as published with the data: it has been reconstructed from the taxon strings. The relatively high amount of records with null or ‘Unknown’ kingdom values shown in figure 10 was actually formed by plant species when examined. However, almost all these records were also lacking any other taxon level above species name. A foreseeable problem with such incomplete data is that it may be difficult to attribute the record to a known taxon concept automatically, especially if the spelling is wrong or the name used is obsolete. This may result in a large number of “orphans”, making it difficult to retrieve records when the dataset of interest is that of a higher taxon such as a family.

On the other hand, automated attribution of taxon concepts may conflict with supplied taxonomies, for those very same reasons (misspellings, older/alternate/”wrong” taxonomies), with a potential for hiding records during a search. This problem has been recognized as general in many taxonomical databases and is not specific of GBIF.ES or GBIF, and is exacerbated when the data of interest to be retrieved are spread across different taxon groups.

Our analysis showed that automated taxon concept attribution as performed at indexing time using the CoL backbone failed in more than 50% of cases for order and class, and was compounded by a low supplier-provided family declaration for the records. This calls for extensive work in the taxonomical backbone(s) of GBIF (perhaps in the form of adopting extensive synonym thesauri or allowing for alternate taxonomies at search time), but that could be useless if the data providers themselves do not supply at least some additional information to restrict the search scope, for example by adapting to an existing taxonomy at least at the family level.

The GBIF.ES dataset contains more than one quarter million distinct data strings and clearly this does not allow for routine manual review and correction of the records supplied by the providers (and that could be extended to the whole GBIF by two orders of magnitude).

When corrected, however, the taxonomy appears as a clear representation of the history of data acquisition. Plants dominate the scene, while less known organisms such as fungi or chromista have a much smaller representation in terms of records. Among animals, the usual suspects dominate (birds, mammals, some insects) disproportionally to their group’s biodiversity. Again then, the taxonomy of records seems to reveal that the dataset may be accurately reflecting the historical distribution of research efforts, personal interests and digitalization opportunities afforded by the biodiversity databases and their hosting institutions.

### Conclusion

In general terms, GBIF.ES shares a large set of ‘good’ biodiversity records. We could detect no critical artifactual patterns or biases in any of the PBD-related fields that could not readily stand out for consideration, and the volume of information made available has the potential to enhance biodiversity research, both locally and globally.

Nevertheless, there are some issues inherent to human data manipulation, from data gathering and sampling to data publishing through digitization processes. Some of those issues might be straightforward to solve, for example, omitting date-related fields with 0 value. Some others might be a bit harder to solve, such as georeferencing records with verbatim location description. And yet some of them might be impossible to solve, like improving the detail level from absent information. A special effort should be made in improving the taxonomy of the records, trying to avoid null levels on taxonomic hierarchies.

If treated properly, the uncertainty these errors represent can be overridden. This is why a constant update of the records and a regular check of their status are highly recommendable in order to keep being a valid and highly useful source of good quality biodiversity data.

As stated earlier, much of the detected error volume could have been reduced by using appropriate data entry protocols at the time of digitization, such as controlled vocabulary and ontologies for taxonomical datasets as suggested by the Biodiversity Information Standards, formerly Taxonomical Databases Working Group (TDWG) (http://www.tdwg.org/). Unfortunately, our analysis also clearly shows that the disparity of resources and protocols using at the multitude of data publishers has allowed for a correspondingly high variety of data input errors that have now become legacy. However, additional problems specifically lie in the mapping of legacy or in-house databases into the DwC exchange standard prior to harvesting by GBIF, and these could be avoided by careful re-design of the mapping protocols taking into account the specifics of each source database.

GBIF nodes (coordination units, national focal points or secretariats of national or thematic biodiversity networks) should pay attention to maintenance of online published data, as they are subject to additions and corrections, and as otherwise they may become obsolete. Another recommendation would be to keep an open mind about provenance of data: collections, observational networks, environmental assessment, etc. as all contribute to the global/total picture.

The findings suggest that overall effectiveness of biodiversity data networks would be significantly improved by recording *ab initio* basic taxo-time-geo referencing (i.e., higher classification, full date, coordinates) even if this goes somewhat beyond the scope of the project at hand.

Some patterns found and explained here may provide some language and basis to develop or refine data cleaning tools. This is especially obvious for the geospatial component.
